# The Envelope Residues E152/156/158 of Zika Virus Influence the Early Stages of Virus Infection in Human Cells

**DOI:** 10.3390/cells8111444

**Published:** 2019-11-15

**Authors:** Sandra Bos, Wildriss Viranaicken, Etienne Frumence, Ge Li, Philippe Desprès, Richard Y. Zhao, Gilles Gadea

**Affiliations:** 1 Université de la Réunion, INSERM U1187, CNRS UMR 9192, IRD UMR 249, Unité Mixte Processus Infectieux en Milieu Insulaire Tropical, Plateforme Technologique CYROI, 94791 Sainte Clotilde, La Réunion, France; sandrabos.lab@gmail.com (S.B.); wildriss.viranaicken@univ-reunion.fr (W.V.); etienne.frumence@univ-reunion.fr (E.F.); philippe.despres@univ-reunion.fr (P.D.); 2 Department of Pathology, University of Maryland School of Medicine, Baltimore, MD 21201, USA; lige_cn@hotmail.com; 3 Department of Microbiology and Immunology, University of Maryland, Baltimore, MD 21201, USA; 4 Institute of Global Health, University of Maryland, Baltimore, MD 21201, USA; 5 Institute of Human Virology, University of Maryland, Baltimore, MD 21201, USA

**Keywords:** flavivirus, Zika virus, envelope protein, glycosylation, fusion loop, viral fusion, cell entry

## Abstract

Emerging infections of mosquito-borne Zika virus (ZIKV) pose an increasing threat to human health, as documented over the recent years in South Pacific islands and the Americas in recent years. To better understand molecular mechanisms underlying the increase in human cases with severe pathologies, we recently demonstrated the functional roles of structural proteins capsid (C), pre-membrane (prM), and envelop (E) of ZIKV epidemic strains with the initiation of viral infection in human cells. Specifically, we found that the C-prM region contributes to permissiveness of human host cells to ZIKV infection and ZIKV-induced cytopathic effects, whereas the E protein is associated with viral attachment and early infection. In the present study, we further characterize ZIKV E proteins by investigating the roles of residues isoleucine 152 (Ile152), threonine 156 (Thr156), and histidine 158 (His158) (i.e., the E-152/156/158 residues), which surround a unique *N*-glycosylation site (E-154), in permissiveness of human host cells to epidemic ZIKV infection. For comparison purpose, we generated mutant molecular clones of epidemic BeH819015 (BR15) and historical MR766-NIID (MR766) strains that carry each other’s E-152/156/158 residues, respectively. We observed that the BR15 mutant containing the E-152/156/158 residues from MR766 was less infectious in A549-Dual™ cells than parental virus. In contrast, the MR766 mutant containing E-152/156/158 residues from BR15 displayed increased infectivity. The observed differences in infectivity were, however, not correlated with changes in viral binding onto host-cells or cellular responses to viral infection. Instead, the E-152/156/158 residues from BR15 were associated with an increased efficiency of viral membrane fusion inside infected cells due to conformational changes of E protein that enhance exposure of the fusion loop. Our data highlight an important contribution of E-152/156/158 residues to the early steps of ZIKV infection in human cells.

## 1. Introduction

Mosquito-borne Zika (ZIKV), dengue (DENV), Yellow fever (YFV) and Japanese encephalitis (JEV) viruses belonging to flavivirus genus (*Flaviviridae* family), are four enveloped RNA viruses of significant public health concern worldwide [1,2,3,4]. Recent ZIKV global outbreaks, with Brazil at the epicentre, highlighted how a previously neglected flavivirus can turn into a severe threat for human health. While human ZIKV infections remained only sporadic and with a limited impact for decades [5,6,7,8], recent outbreaks revealed that ZIKV caused clusters of severe congenital and neurological abnormalities in infants and peripheral nervous system impairments in adults [9,10,11,12]. Considering the dramatic increase of severe human cases, strategies to efficiently control this virus, either in terms of antiviral therapies or vaccines, are urgently needed and a granted requirement for more extensive studies.

Flaviviruses contain a genomic single-stranded positive RNA encoding a single large polyprotein that is subsequently cleaved by cellular and viral proteases into three structural proteins (C, prM/M and E) and seven nonstructural proteins (NS1 to NS5). The latter are responsible for virus replication, assembly and escape from host immune system, while structural proteins form the viral particle surrounding genomic viral RNA. Among structural proteins, the E protein is responsible for viral entry into host cells. Viral E protein first binds to cellular attachment factors and receptors, leading to virion internalisation primarily through a clathrin-mediated endocytic pathway [13]. In endosomes, fusion of viral and cellular membranes occurs after E protein conformational changes triggered by low pH [14]. The E protein peptide chain folds into three distinct domains: a central ß-barrel (domain EDI), an elongated dimerization region (domain EDII), which includes the fusion loop, and a C-terminal, immunoglobulin-like module (domain EDIII) [15]. Most flavivirus E proteins are post-translationally modified by addition of a single N-linked oligosaccharide on residue N-154 located within the EDI-loop [16]. Flavivirus E proteins represent one of the key determinants for viral pathogenesis. Flavivirus envelope supports virus tropism and single amino-acid changes can redirect virus tropism [17]. Flavivirus E proteins also represent a major target for neutralizing antibodies but, at the same time, can be involved in enhancement/cross-reactivity of reactive antibodies [18,19,20,21].

Recently, our studies on chimeric ZIKV clones between an epidemic Brazilian strain of ZIKV BeH819015 (hereafter called BR15) and a historical African strain MR766 highlighted an important role of two structural proteins prM/M, and E in ZIKV ability to infect human cells [16,22,23,24]. We further showed that they contribute to the initiation of viral infection. Analysis of chimeric viruses indicated that C-prM region plays a role in triggering cell death by ZIKV and E protein is associated with viral attachment to host cells during early infection [23,24]. Flavivirus E proteins usually contain two *N*-glycosylation sites at position E-56 and E-154. The first site is lacking in ZIKV E protein and contribution of the second site in ZIKV viral cycle, including in the mosquito vector, has been recently highlighted [25]. N-linked glycosylation of the E protein was shown to be an important determinant of ZIKV virulence in a mouse model of viral encephalitis [22,26]. In invertebrate vectors, ZIKV bearing an unglycosylated E protein was attenuated in its capacity to replicate in *Aedes aegypti* [27]. Although a *N*-glycosylation site is highly conserved among flaviviruses, which suggests of its biological importance, E proteins could remain unglycosylated as it has been observed in some ZIKV strains. To date, the exact mechanism by which the *N*-glycosylation motif region of the E protein contributes to ZIKV infectivity still remains elusive. In the present study, we further characterised structural protein contribution in ZIKV infectivity by focusing on the ZIKV E protein. Our goal was to determine whether three E residues—Ile152, Thr156, and His158 (hereafter called as E-152/156/158 residues)—which surround the Asn154 composing the *N*-glycosylation site NDT, may have any effect on ZIKV’s ability to infect human cells.

## 2. Materials and Methods

### 2.1. Cells and Reagents

Vero cells (CCL-81, ATCC, Manassas, VA, USA), A549-Dual™ cells (a549d-nfis, InvivoGen, San Diego, CA, USA) and human embryonic kidney HEK-293 cells (CRL-1573, ATCC, Manassas, VA, USA) were cultured at 37 °C under a 5% CO_2_ atmosphere in MEM medium, supplemented with 5% to 10% heat-inactivated foetal bovine serum (FBS). A549-Dual™ (A549^DUAL^) cells were maintained in growth medium supplemented with nonessential amino acids, 10 µg·mL^−1^ blasticidin and 100 µg·mL^−1^ zeocin (InvivoGen, San Diego, CA, USA). Chloroquine phosphate was purchased from Sigma-Aldrich (Saint-Louis, MO, USA). Rat antibody specifically raised against ZIKV E protein Domain III was developed *in-house* and used in immunoblot with reducing conditions [28]. Mouse anti-pan flavivirus envelope E protein monoclonal antibody (mAb) 4G2 was purchased from RD Biotech (Besancon, France) and used in immunoblot with nonreducing conditions. Horseradish peroxidase-conjugated anti-rabbit and anti-mouse antibodies were purchased from Vector Laboratories (Burlingame, CA, USA).

### 2.2. Design of ZIKV Molecular Clones

ZIKV molecular clones (MR766, GenBank accession number LC002520, and BR15, GenBank accession number KU365778) were designed and produced according to the Infectious Subgenomic Amplicon method as previously described [23,29,30]. To introduce BR15 E-152/156/158 residues into MR766 (MR766^E-152I/156T/158H^), we used mutagenesis primers (forward primer: 5′-ggctcccagcacagtgggatgatcgttaatgacacaggacatgaaactg-3′ and reverse primer: 5′-cagtttcatgtcctgtgtcattaacgatcatcccactgtgctgggagcc-3′) to generate two overlapping fragments Z1^MR766-E-MUT1^ and Z1^MR766-E-MUT2^ from the Z1^MR766^ fragment encoding the MR766 structural proteins in which encoding region of the E protein received the IVNDTGH motif (amino acids 152 to 158) from BR15. To generate BR15^E-152T/156I/158Y^, a new Z1^BR15-E-I152T/T156I/H158Y^ fragment was synthesised in which the sequence was modified so that encoding region of the E protein received the TVNDIGY motif (amino acids 152 to 158) from MR766. Synthetic genes were cloned into plasmid pUC57 by GeneCust (Boynes, France). Fragments were amplified by PCR from their respective plasmids using a set of primer pairs that was designed so that fragments overlapped with each other of about 30 to 50 nucleotides.

### 2.3. Recovering of Molecular Clones BR15^E-152T/156I/158Y^ and MR766^E-152I/156T/158H^

Molecular clones were produced as previously described [23,29]. Briefly, purified PCR fragments were electroporated into Vero cells. After 5 days, cell supernatants were recovered usually in absence of cytopathic effect and used to infect fresh Vero cells in a first round of amplification (P1). Viral clones were recovered at the onset of cytopathic effect and amplified for another round on Vero cells to produce a second round of amplification (P2), which was used for described studies. To produce MR766^E-152I/156T/158H^ and BR15^E-152T/156I/158Y^ mutant viral clones, Vero cells were respectively electroporated with PCR fragments Z1^MR766-E-MUT1^, Z1^MR766-E-MUT2^, Z2^MR766^, Z3^MR766^, and Z4^MR766^ and with Z1^BR15-E-MUT^, Z2^BR15^, Z3^BR15^, and Z4^BR15^. Recovered mutant viruses MR766^E-152I/156T/158H^ and BR15^E-152T/156I/158Y^ respectively consist of viral sequence of MR766 in which E-152/156/158 residues of BR15 ZIKV strain were introduced and viral sequence of BR15 in which E-152/156/158 residues were replaced with its counterpart from MR766 ZIKV strain.

### 2.4. Plaque-Forming Assay

Viral titres were determined by a standard plaque-forming assay as previously described with minor modifications [31]. Briefly, Vero cells grown in a 48-well culture plate were infected with serial tenfold dilutions of virus samples for 2 h at 37 °C, and then incubated with 0.8% carboxymethylcellulose (CMC) for 4 days. Cells were fixed with 3.7% formaldehyde (FA) in PBS and stained with 0.5% crystal violet in 20% ethanol. Viral titres were expressed as plaque-forming units (PFU) per mL (PFU·mL^−1^).

### 2.5. Quantification of Viral Stocks

Zika virus samples were analysed by titration on Vero cells while genomic viral RNA was quantified by RT-qPCR, as previously described [23]. Briefly, viral RNA was extracted from virus particles using QIAmp Kit (Qiagen, Hilden, Germany). PCR standard curve used for quantification of ZIKV copy numbers was obtained with a pUC57/ZIKV-E amplicon plasmid containing a synthetic cDNA encompassing nucleotides 961 to 1301 of genomic RNA (MR766). The pair of ZIKV E primers was used to equally amplify pUC57/ZIKV-E amplicon and cDNA encompassing nucleotides 1046 to 1213 from genomic RNA of ZIKV molecular clones used in this study.

### 2.6. Immunoblot Assay

Cell lysates were performed in RIPA lysis buffer or buffer A (cell fractionation). All subsequent steps of immunoblotting were performed as previously described [32,33]. Primary antibodies were used at 1:500 dilutions. Anti-mouse immunoglobulin-horseradish peroxidase and anti-rat immunoglobulin-horseradish peroxidase conjugates were used as secondary antibodies (dilution 1:2000). Blots were revealed with ECL detection reagents (Amersham, Little Chalfont, United Kingdom).

### 2.7. Flow Cytometry Assay

A549-Dual™ cells were grown on six-well plates at 5 × 10^5^ cells per well and infected at a multiplicity of infection (MOI) of 1. Infected cells were harvested and fixed with 3.7% formaldehyde in PBS for 20 min, permeabilized with 0.1% Triton X-100 in PBS for 4 min and then blocked with PBS-BSA for 10 min. Cells were stained with anti-E mAb 4G2 (1:1000) for 1 h. Antigen staining was visualized with goat anti-mouse Alexa Fluor 488 IgG (1:1000) for 20 min. Cells were subjected to a flow cytometric analysis using a CytoFLEX flow cytometer (Beckman Coulter, Brea, CA, USA). The percentage of positive cells was determined using FlowJo software (version 10, Tree Star, Inc., Ashland, OR, USA).

### 2.8. RT-qPCR

Total RNA including genomic viral RNA was extracted from cells (Qiagen, Hilden, Germany) and reverse transcription was performed using 500 ng of total RNA, random hexamer primers (intracellular viral RNA) or E reverse primer (virus particles) and moloney mouse leukemia virus reverse transcriptase (Life Technologies, Carlsbad, CA, USA) at 42 °C for 50 min. Quantitative PCR was performed on a CFX96 qPCR System (Bio-Rad, Hercules, CA, USA). Briefly, 10 ng of cDNA was amplified using 0.2 μM of each primer and 1X GoTaq Master Mix (Promega, Madison, WI, USA). When appropriate, data were normalised to the internal standard GAPDH. For each single-well amplification reaction, a threshold cycle (Ct) was calculated using the CFX96 qPCR program (Bio Rad, Hercules, CA, USA) in the exponential phase of amplification. Relative changes in gene expression were determined using the 2ΔΔCt method and reported relative to the control. Primers used in this study are listed in [31]. ZIKV E primers were designed to match both MR766-NIID and BeH819015 sequences (forward 5-gtcttggaacatggagg-3′ and reverse 5′-ttcaccttgtgttgggc-3′).

### 2.9. Virus Binding Assay

Cells were cultured at subconfluent density in 24-well plates. Cell monolayers were washed in cold PBS and cooled at 4 °C for at least 20 min in presence of cold MEM supplemented with 2% FBS. Pre-chilled cells were incubated at 4 °C with ZIKV at MOI of 1 in 1.5 mL of cold MEM supplemented with 2% FBS. After 1 h of incubation, virus inputs were removed and cells were washed with cold MEM supplemented with 2% FBS. Total cellular RNA was extracted using the RNeasy kit (Qiagen, Hilden, Germany) and RT-qPCR analysis on viral RNA was performed using primers for ZIKV E gene as described above.

### 2.10. Fusion Assay

Cells were cultured at subconfluent density in 12-well plates. Cell monolayers were cooled at 4 °C for at least 20 min in presence of cold MEM supplemented with 10% FBS. Pre-chilled cells were incubated at 4 °C with ZIKV at MOI of 1 in 1 mL of cold MEM supplemented with 10% FBS. After 1-h incubation, cells were shifted to 37 °C for another hour. Chloroquine was added to culture medium at 100 µM for a 2-h period. Next, culture medium was then replaced to avoid drug cytotoxic effects. Cells were harvested 30 h post temperature shifting for RNA extraction. Total RNA was subjected to RT-qPCR analysis as described.

### 2.11. Cytotoxicity Assay

Cell damages were evaluated by measuring lactate dehydrogenase (LDH) release. Supernatants of infected cells were recovered and subjected to CytoTox 96^®^ nonradioactive cytotoxicity assay (Promega, Madison, WI, USA) according to manufacturer instructions. Absorbance of converted dye was measured at 490 nm using a microplate reader (Tecan, Mannedorf, Switzerland). Results of LDH activity in cell supernatants are presented with subtraction of values from mock-infected cells.

### 2.12. Measurement of the IFN-β Pathway Activation

Activation of the Interferon regulatory factors (IRF) pathway was monitored by measuring *Lucia* luciferase activity in A549-Dual™ cells. It was evaluated using QUANTI-Luc substrate (InvivoGen, San Diego, CA, USA) according to manufacturer’s recommendations. IRF-induced luciferase levels were quantified using a FLUOstar Omega Microplate Reader (BMG LABTECH, Offenburg, Germany). Results are presented with subtraction of values from mock-infected cells.

### 2.13. TMD2-M/E Expression

To express recombinant E proteins from ZIKV in mammalian cells, TMD2-prM (TransMembrane Domain II) and E genes from MR766 and BR15, as well as a mutant BR15 bearing residues E-152 to E-158 from MR766, were synthesised by GeneCust (Boynes, France). Recombinant proteins comprised aa 275 to aa 775 of polyproteins, which correspond to the very end of prM protein (TMD2, used as signal peptide for E proteins) and the entire E protein. As Flavivirus prM protein plays a role of chaperone to ensure the proper folding of the E protein, we expected that viral chaperone activity eviction would have exacerbate differences in E protein folding [34]. Modifications to optimize viral E protein expression in human cells were done on original protein sequences. Then, mammalian codon-optimised sequences coding for TMD2-prM and E proteins were cloned into the *NheI* and *NotI* restriction sites of the pcDNA3.1(-) plasmid to generate pMR766, pBR15 and pBR15^E-MUT^, respectively. Each plasmid was transfected in human HEK-293T cells using lipofectamine 3000 according to manufacturer’s instructions.

### 2.14. Cell Fractionation

Cells were washed with PBS and lysed at a concentration of 1 × 10^4^ cells per µl in protein separation buffer A (0.2% Triton X-100, 50 mM Tris-HCl pH 7.5, 150 mM NaCl, 2.5 mM EDTA) [32]. As misfolded proteins aggregate and become resistant to Triton X-100 solubilisation [32,35], Triton X-100-insoluble fraction was separated by centrifugation at 3400 *g* for 10 min. Pellets were enriched in misfolded proteins. Samples were analysed by immunoblot. Loading was normalised by the number of lysed cells. Band intensities were determined with ImageJ software (version 1.50i, NIH, Washington, WA, USA, 2016) and soluble/insoluble ratios calculated.

### 2.15. Statistical Analysis

All values are expressed as mean ± SD of at least two independent experiments. Comparisons between different treatments were analysed by a one-way or two-way ANOVA tests as deemed appropriate. Values of *p* < 0.05 were considered statistically significant for a post-hoc Tukey′s test. All statistical tests were done using the software GraphPad Prism (version 8.01, GraphPad Software, La Jolla, CA, USA).

## 3. Results

### 3.1. Characterization of Mutant ZIKV Molecular Clones

**Figure 1:**
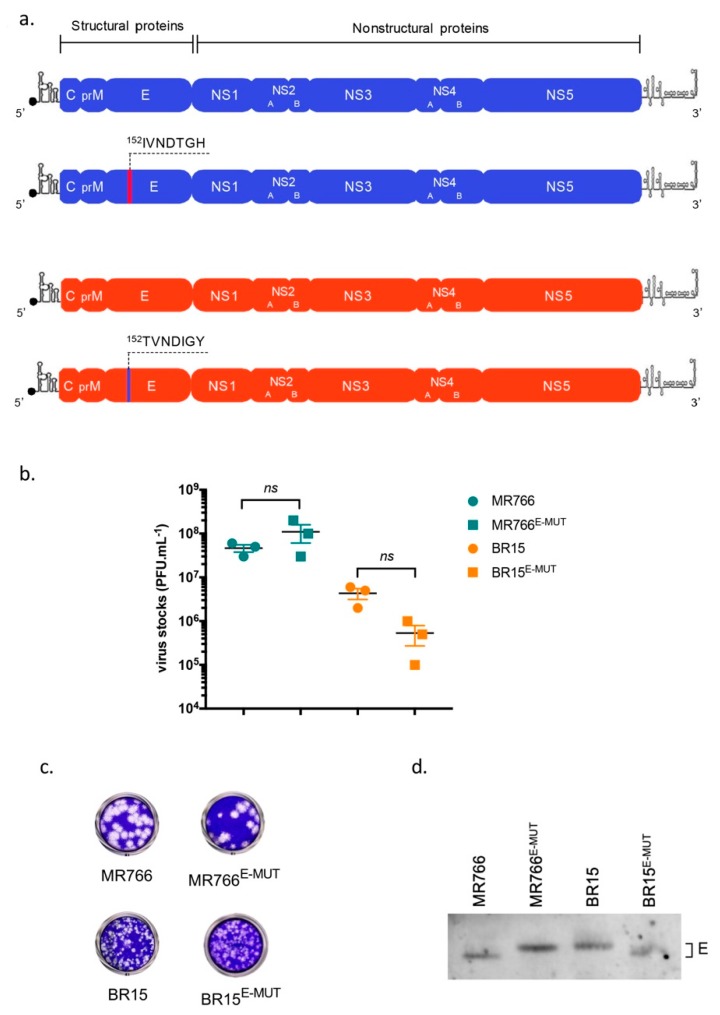
ZIKV mutant molecular clones. In (**a**), schematic representation of mutant viral clones BR15^E-MUT^ and MR766^E-MUT^ and their respective parental clones. In (**b**), histograms showing viral titres. Values represent means and standard errors of three independent experiments. In (**c**), examples of infectious plaques developed for BR15^E-MUT^ and MR766^E-MUT^, and parental clones, after plaque-forming assay on Vero cells. In (**d**), Vero cells were infected with parental and mutant molecular clones (MR766 and BR15) at a MOI of 1. 24 h post-infection (hpi), cells were then lysed and subjected to an immunoblot, in non-reducing conditions. Anti-ZIKV EDIII immunoblot shows differences of electrophoretic mobility associated with residue mutations.

To determine the contribution of E-152/156/158 residues in ZIKV E protein functions, we generated two mutant molecular clones: MR766^E-152I/156T/158H^, hereafter called MR766^E-MUT^, in which E-152/156/158 residues of BR15 epidemic strain were introduced, and BR15^E-152T/156I/158Y^, hereafter called BR15^E-MUT^, in which E-152/156/158 residues were replaced with their counterparts from the MR766 historical African strain (Figure 1a). Genomes were assembled using the infectious subgenomic amplicon method [29]. Briefly, Vero cells were electroporated with overlapping fragments, in which appropriate mutations have been previously introduced. The two recovered clones were viable and twice amplified on Vero cells. Titres of P2 working viral stocks were determined in Vero cells and were ranging from 5 × 10^5^ to 1 × 10^8^ PFU·mL^−1^ (Figure 1b). MR766^E-MUT^ and BR15^E-MUT^ gave plaque morphologies that resembled those of respective MR766 and BR15 parental clones (Figure 1c), which is in agreement with previously published data [27]. In addition, we confirmed that the introduced mutations affected the electrophoretic mobility of ZIKV E proteins, suggesting that E-152/156/158 residues from BR15 E protein might enable its glycosylation (Figure 1d + Figure S1).

### 3.2. Residues E-152/156/158 from BR15 Potentiate Viral Infectivity

We first analysed infectivity of P2 virus stocks as described above. Particle-to-PFU ratios obtained from parental clones were around 900–1000 (Table 1), which is consistent with our previous observations [23]. We then analysed particle-to-PFU ratios of the two E mutant clones. Addition of residues E-152/156/158 from BR15 to MR766 resulted in a 2.3-fold decrease in the particle-to-PFU ratio. In contrast, when residues E-152/156/158 from MR766 were introduced to BR15, the particle-to-PFU ratio was markedly increased with more than ten-folds. These results suggest that residues E-152/156/158 from BR15 potentiate virion infectivity.

### 3.3. Alteration of Residues E-152 to E-158 of ZIKV E Protein Does Not Affect Virus Binding to Host Cells but May Affect Virus Progeny Production

**Figure 2:**
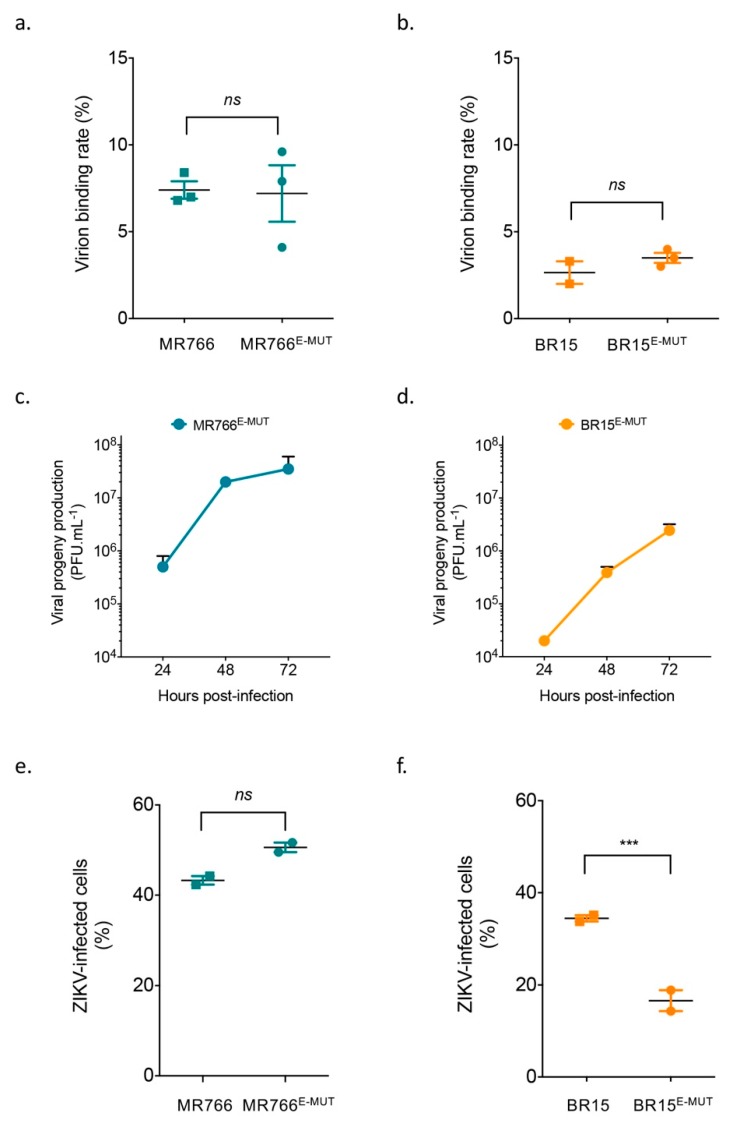
Analysis of virus binding and viral growth in A549-Dual™. In (**a**) and (**b**), for virus binding assays, cells were incubated with viral clones at the MOI of 1 for 1 h at 4 °C. The number of virus particles bound to cell surface was measured by RT-qPCR. Values represent means and standard errors of three independent experiments. In (**c**) and (**d**), A549-Dual™ were infected with BR15^E-MUT^ and MR766^E-MUT^ at MOI of 1. Infectious virus released into the supernatants of infected A549-Dual™ cells were quantified at 24, 48 and 72 hpi. Error bars represent standard errors of at least two independent experiments. In (**e**) and (**f**), A549-Dual™ were infected with BR15^E-MUT^ and MR766^E-MUT^ and parental clones at MOI of 1. Percentages of ZIKV-infected cells were determined at 48 h by flow cytometry using anti-E mAb 4G2 as primary antibody. Error bars represent standard errors of two independent experiments in duplicates. *ns*: not significant, ***: *p* value < 0.001

We previously showed that historical and epidemic ZIKV strains display differences in their abilities to bind host cells, leading to differences in cell susceptibility to infection (18, 19). Here, we further investigated the ability of the described mutant clones to bind onto A549-Dual™ cells. Virus binding assays were performed and analysed by RT-qPCR to determine virus particle binding onto cell surface after an incubation period of 1 h. Panels A and B show no difference between mutant clones and their respective parental clones (Figure 2). These results contrast with other studies in mosquito cells [27], suggesting that viral receptors may vary between vertebrate and invertebrate cells. These data suggest that alteration of residues E-152 to E-158 of ZIKV E protein does not affect virus bindings to A549-Dual™ cells.

Instead, these results suggest that E-152/156/158 residues might influence ZIKV progeny production. Indeed, the progeny production of MR766^E-MUT^ was modestly but reproducibly increased in comparison to that of MR766 (3 × 10^7^ PFU·mL^−1^ vs. 1 × 10^7^ PFU·mL^−1^) at 72 hpi [23]. Conversely, kinetics of the BR15^E-MUT^ progeny production were strongly altered compared to BR15 (2 × 10^6^ PFU·mL^−1^ vs. 4 × 10^7^ PFU·mL^−1^) at 72 hpi, respectively [23]. Differences in progeny production were observed at as early as 24 hpi. Similar differences were also seen on the percentages of the infected cell at 48 hpi. Taken together, these results indicate that E-152/156/158 residues from BR15 potentiate viral infectivity, independently of the virus binding to host A549-Dual™ cells.

### 3.4. Mutations at E-152/156/158 Residues Have No Effect on ZIKV-Induced Cell Death or Interferon Pathways

**Figure 3:**
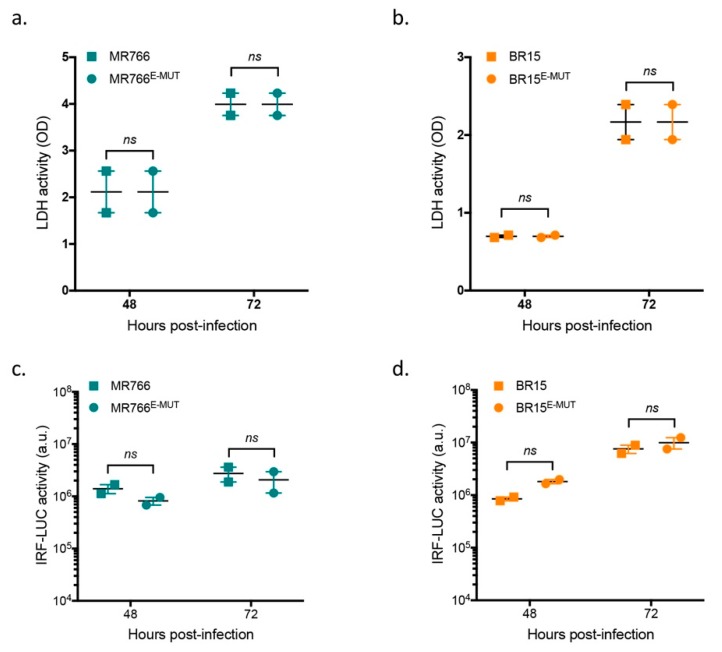
Analysis of infection-induced cell death and immune responses. A549-Dual™ were infected with BR15^E-MUT^ and MR766^E-MUT^ and parental clones at MOI of 1. In (**a**) and (**b**), LDH activity was measured at 48 and 72 hpi respectively. Values represents mean and standard errors of two independent experiments in triplicates. In (**c**) and (**d**), analysis of IRF pathway activation in response to viral infection. Activity of secreted *Lucia* luciferase was measured using QUANTI-Luc substrate at 48 and 72 hpi. Results are expressed as raw data of luminescence arbitrary units. Error bars represent standard errors of two independent experiments in triplicates. *ns*: not significant.

To determine whether differences described with the mutant viruses were associated with specific host-cell responses, we first analysed virus-induced cell death at 48 h and 72 h post-infection. No difference in cytotoxicity measured by LDH release was observed between wild-type and mutant viruses (MR766 and BR15) (Figure 3, panels a and b). We then took advantage of the properties of A549-Dual™ cells to test whether mutant viruses can trigger different host cell innate immunity. A549-Dual™ cells were derived from A549 cells by stable integration of two reporter genes: *SEAP* gene (Secreted Embryonic Alkaline Phosphatase) and *Lucia* luciferase gene under the respective transcriptional control of an IFN-β minimal promoter, which is fused to NF-κB binding sites or an ISG54 minimal promoter in conjunction with interferon-sensitive response elements. We examined possible activation of the IRF pathway by monitoring production of *Lucia* luciferase at 48 dpi and 72 hpi. Similar responses were observed in both wild-type and mutant clones (Figure 3, panels c and d). The NF-κB pathway was not investigated, as we showed previously that this pathway is not activated upon ZIKV infection [23]. These results indicate that differences in the mutant virus properties could not be explained by specific host-cell responses, which are consistent with our previous observations suggesting a link between host-cell responses and ZIKV nonstructural proteins [23].

### 3.5. E-152/156/158 Residues from BR15 Facilitate Viral Fusion

**Figure 4:**
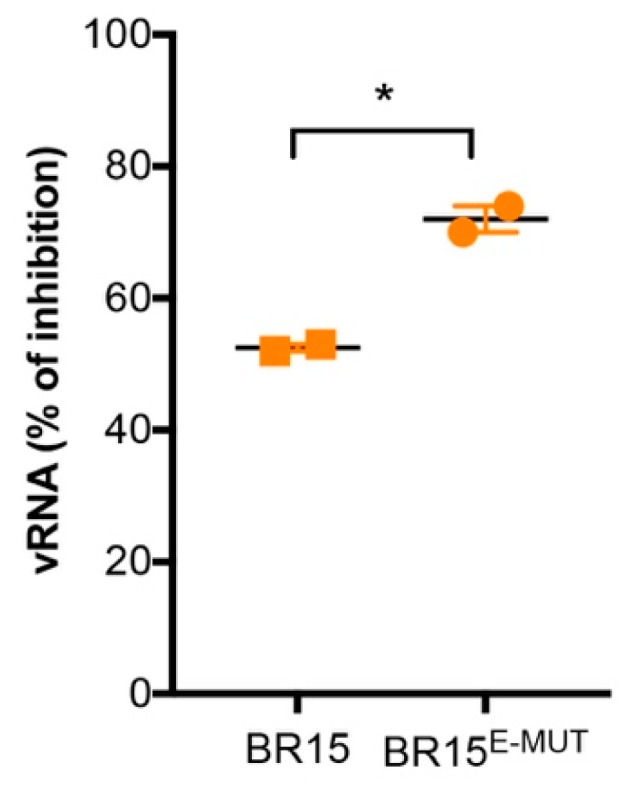
Viral fusion in A549-Dual™ cells. Pre-chilled cells were incubated at 4 °C with ZIKV at MOI of 1. After 1-h incubation, cells were shifted to 37 °C. Chloroquine was then added to the culture medium. Viral RNA was measured by RT-qPCR 30 h at 37 °C. Error bars represent standard errors of two independent experiments. *: *p* value < 0.1

We showed earlier that E-152/156/158 residues from BR15 have a growth advantage without apparent association with cellular attachment (Figure 2) or specific host-cell responses (Figure 3). We then studied viral fusion to test whether it could explain the observed growth advantage. Viral fusion of flaviviruses is commonly triggered from endosomes upon low-pH by a series of molecular changes within the E protein, resulting in the release of the nucleocapsid into cell cytoplasm. Chloroquine, a 4-aminoquinoline, is a weak base that inhibits endosome acidification and consequently restricts viral replication of many viruses through inhibition of pH-dependent steps. Recently, chloroquine was shown to inhibit Zika virus infection in different cellular models [36,37]. As BR15 and BR15^E-MUT^ showed significant differences in the percentage of infected cells (Figure 2f), we decided to focus on these two molecular clones for the following virus fusion experiments. We treated A549-Dual™ cells infected with BR15 or BR15^E-MUT^ with 100 µM of chloroquine 1 hpi for 2 h and then cells were moved back to regular medium. Intracellular viral RNA was quantified 30 hpi by RT-qPCR. BR15^E-MUT^ fusion was significantly restricted by chloroquine treatment compared to that of BR15 (Figure 4). These data suggest that E-152/156/158 residues from BR15 favour viral fusion with host-cell membranes.

### 3.6. E-152/156/158 Residues from BR15 Favour Conformational Changes within the Fusion Loop

**Figure 5:**
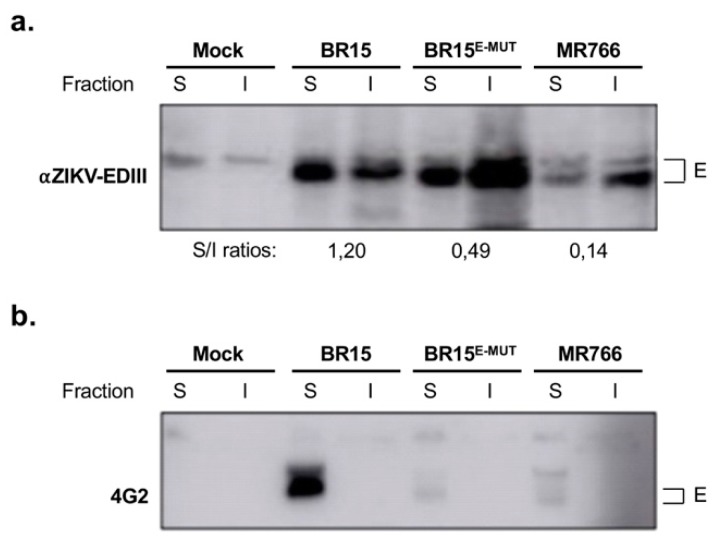
Conformational changes induced by residues E-152/156/158 of ZIKV E protein. In (**a**) and (**b**), HEK-293T cells were transfected with TMD2-prM/E constructs and antibiotics that were selected to raise stable cell lines. Cells were harvested and proteins extracts subjected to a fractionation. Protein fractions were immunoblotted with anti-ZIKV EDIII (**a**) or anti-E 4G2 (**b**) antibodies. S, soluble proteins; I, insoluble proteins. Band intensities were determined with ImageJ software and S/I ratios were calculated. Apparent discrepancies with cytometry experiments (Figure 2) regarding antibody reactivity are explained by experimental and recombinant protein overexpression versus viral infection conditions.

As virus fusion with host cells is highly dependent on conformational changes of the E protein triggered at low-pH, we hypothesize that the reduced fusion we observed with the mutant molecular clone BR15^E-MUT^ bearing E-152/156/158 residues from MR766 could be the consequence of conformational differences between E proteins of the two molecular clones. In order to test this hypothesis, BR15, BR15^E-MUT^ and MR766 sequences coding for TMD2-prM/E were codon-optimised for expression in mammalian cells and cloned into a pcDNA3.1 vector. HEK-293T cells were transfected with different plasmids and positive cells were selected with antibiotics. The resulting stable cell lines were fractionated to evaluate the capacity of recombinant E proteins to fold properly, insolubility been a hallmark of misfolded proteins [32,35]. Resulting fractions were subjected to an immunoblot analysis. We first used a rat antibody developed in-house, specifically raised against E protein domain EDIII [28]. Figure 5a revealed that BR15^E-MUT^ and MR766 TMD2-prM/E overexpression resulted in a greater E protein propensity to accumulate in insoluble fractions than that of BR15 overexpression, as shown by inversion of soluble/insoluble ratios. Interestingly, differences observed between BR15 and mutant BR15 TMD2-prM/E suggested that ZIKV E proteins bear different conformations that only depend on E-152/156/158 residues. To verify these observations, the same samples were immunoblotted using a 4G2 monoclonal antibody, which recognises a highly conserved fusion loop sequence of most flaviviruses. As shown in Figure 5b, 4G2 monoclonal antibody strongly reacts against E protein from BR15, whereas we could barely detect any signal with the two E proteins bearing E-152/156/158 residues from MR766. Preliminary in silico modelling of ZIKV E proteins suggest that changes in the glycosylation motif could affect structure of the glycosylation and fusion loops as well as interactions with surrounding residues (Figure S2) and surface hydrophobicity (not shown). These data confirm that E-152/156/158 residues in the EDI domain support conformational changes on the ZIKV E protein, which could be detected in the fusion loop of EDII domain. Finally, these results suggest that conformational changes occur in BR15 E protein upon mutation of E-152/156/158 residues.

## 4. Discussion

The role of structural proteins in determining infectivity of human cells to ZIKV infection has been previously reported [23]. Zika viruses of historical or epidemic strains display differences in their abilities to bind host cells leading to differences in cell susceptibility to infection. To further characterise biological properties of contemporary ZIKV strains, which have been associated with recent epidemics and severe forms of human disease, we investigated the role of E-152/156/158 residues of the envelope protein, residues located around a unique *N*-glycosylation site (E-154) in viral entry into host cells. Our mutagenesis data showed that E-152/156/158 residues are responsible for the differences we observed in the infectivity of the virus and progeny production kinetics, without affecting viral attachment and host-cell responses. Further characterisations identified the E protein conformational changes in the fusion loop, supported by E-152/156/158 residues, as a major event in virus fusion and release of viral RNA into cell cytoplasm.

The first step in viral entry pathway involves nonspecific viral binding to cellular attachment factors. Negatively charged glycosaminoglycans, which are abundantly expressed on numerous cell types, are considered as low-affinity attachment factors by flaviviruses. These interactions serve to concentrate viruses on the cell surface and are mediated by the EDIII domain of E proteins [38]. Our data demonstrate that E-152/156/158 residues of ZIKV E protein do not influence virus binding, which suggests that domain EDIII conformation is not strongly affected by E-152/156/158 residues. We conclude that this initial step of ZIKV entry into cells does not depend on E-152/156/158 residues of the E protein.

Viral particle internalisation could occur through distinct routes, including clathrin-mediated endocytosis or non-classical clathrin-independent endocytic pathways. These distinct entry modes depend not only on host cells but also on viral serotype or strain [39]. Despite these differences in the internalisation process, genome release into the cytoplasm always occurs through E protein-mediated membrane fusion [40]. The low-pH environment within endosomes triggers a series of molecular changes within flavivirus E protein resulting in fusion of viral membrane with endosomal membrane and subsequent release of the nucleocapsid into cell cytoplasm [13,14]. The initial step in membrane fusion involves protonation-dependent disruption of E protein rafts at viral surface, resulting in conformational changes and formation of a fusion pore from which the nucleocapsid is released into the cytosol. Evidences suggested that the glycan loop modulates the overall Flavivirus E protein conformation and, specifically, fusion loop exposure [41,42]. Sevvana and colleagues proposed that, given the close proximity of ZIKV glycan and fusion loops, any interaction of the glycan loop with a receptor on a host-cell surface might promote exposure of the fusion loop and facilitate formation of fusogenic trimers leading to membrane fusion [43]. Another study based on antibody neutralizing activities demonstrated that residues surrounding ZIKV E protein glycan regulate virus antigenicity [44]. In a previous study, we also evaluated immunogenicity of a chimeric viral clone ZIKBeHMR-2, in which the region encoding envelope proteins of MR766 African strain backbone was replaced with its counterpart from BeH819015 epidemic strain [45,46]. Amino-acid substitutions I152T, T156I, and H158Y were introduced in the glycan loop of the E protein, making chimeric ZIKBeHMR-2 a nonglycosylated virus. Those results suggest that, rather than just determining the glycosylation, amino-acid residues at position 152, 156 and 158 play a pivotal role on accessibility of neutralizing antibody epitopes on mature virus particles. In this study, we demonstrated that changes in the glycan loop can modulate accessibility to the fusion loop. Although these observations were made independently of the presence of a glycan, it is conceivable that E protein *N*-glycosylation could provide another level of regulation on the access of the fusion loop. The role of *N*-glycosylation on ZIKV E protein has also been investigated using pseudoviral particles, showing that reduced infectivity was observed with mutant viral particles lacking the N-glycan [47]. Altogether, these studies suggest that conformational changes induced at the glycan loop most probably modulate fusion loop exposure and subsequent fusion of viral and cellular membranes, which strongly supports our observations.

One interesting finding from this study is that chloroquine treatment results in less BR15^E-MUT^ entries than it does for BR15. This finding suggests that E-152/156/158 residues of BR15 increase pH sensitivity of E protein. To generate BR15^E-MUT^, its sequence was modified so that the coding region of the E protein IVNDTGH (amino acids 152 to 158) in BR15 was replaced with TVNDIGY motif from MR766, meaning that not only *N*-glycosylation motif was abrogated but also that histidine E-158 was changed into a tyrosine. Histidine residues have been described as pH sensors in flavivirus membrane fusion [48]. Fusion initiation is crucially dependent on protonation of conserved histidine residues at the interface between domains EDI and EDIII of E protein, leading to dissolution of domain interactions and to fusion peptide exposure. Given the fusion differences we observed between wild-type and mutant BR15 molecular clones, further investigations on histidine E-158 protonation are required to determine its exact contribution to membrane fusion.

Finally, our analysis of virus inocula generated in Vero cells showed differences in particle-to-PFU ratios indicating that E-152/156/158 residues of BR15 E protein facilitate release of more infectious particles. In addition, we demonstrated with recombinant proteins that E-152/156/158 residues of epidemic ZIKV E protein also facilitate production of more soluble proteins. These results are supported by works of Mossenta and colleagues [47]. Whether these observations are due to conformational changes occurring during virion production remains undetermined. However, in a study on flavivirus cross-reactive epitopes, Crill and Chang suggested that close packing of fusion peptide against its subunit partner and glycan on the upper surface protects the fusion loop from irreversible pH-induced conformational changes during maturation and secretion [49]. All these observations suggest that E-152/156/158 residues of epidemic ZIKV E protein could also be an advantage during virion maturation process.

Altogether, our data indicate that the envelope residues E152/156/158 of Zika virus influence early stages of virus infection in human cells. This study highlights the importance of E-152/156/158 residues in ZIKV biology and specifically in their roles in supporting viral fusion. These new findings could potentially help to design innovative strategies for future ZIKV infection control.

## Figures and Tables

**Table 1:** Table showing particle-to-PFU ratios. Viral RNA extracted from viral stock P2 were subjected to quantification by RT-qPCR using E primers. Obtained Ct values were plotted in a standard curve (serial dilutions of plasmid copies) in order to get the number of viral RNA molecules per mL. These results were compared to viral stock quantifications by standard plaque-forming assay, which then gave the particle-to-PFU ratios, also named vRNA-to-PFU ratios. Values represent means and standard errors of two to four independent experiments.

VIRUS STOCK.	PARTICLE-TO-PFU RATIO	*p* VALUES (E-MUT vs. WT)
MR766	997 ± 36	
MR766^E-MUT^	419 ± 87	<0.05
BR15	918 ± 91	
BR15^E-MUT^	11 237 ± 720	<0.001

## Supplementary Materials

The following are available online at https://www.mdpi.com/2073-4409/8/11/1444/s1, Figure S1: Tunicamycin treatment of Vero cells infected with ZIKV mutant molecular clones, Figure S2: structures of ZIKV E wild-type and mutant proteins.
