# Supplementary material for: The Envelope Residues E152/156/158 of Zika Virus Influence the Early Stages of Virus Infection in Human Cells
Source: Cells. 2019 Nov 15;8(11):1444. doi: 10.3390/cells8111444 (PMC6912530; doi:10.3390/cells8111444)
Supplement: Supplementary file 1 [file cells-08-01444-s001.pdf]

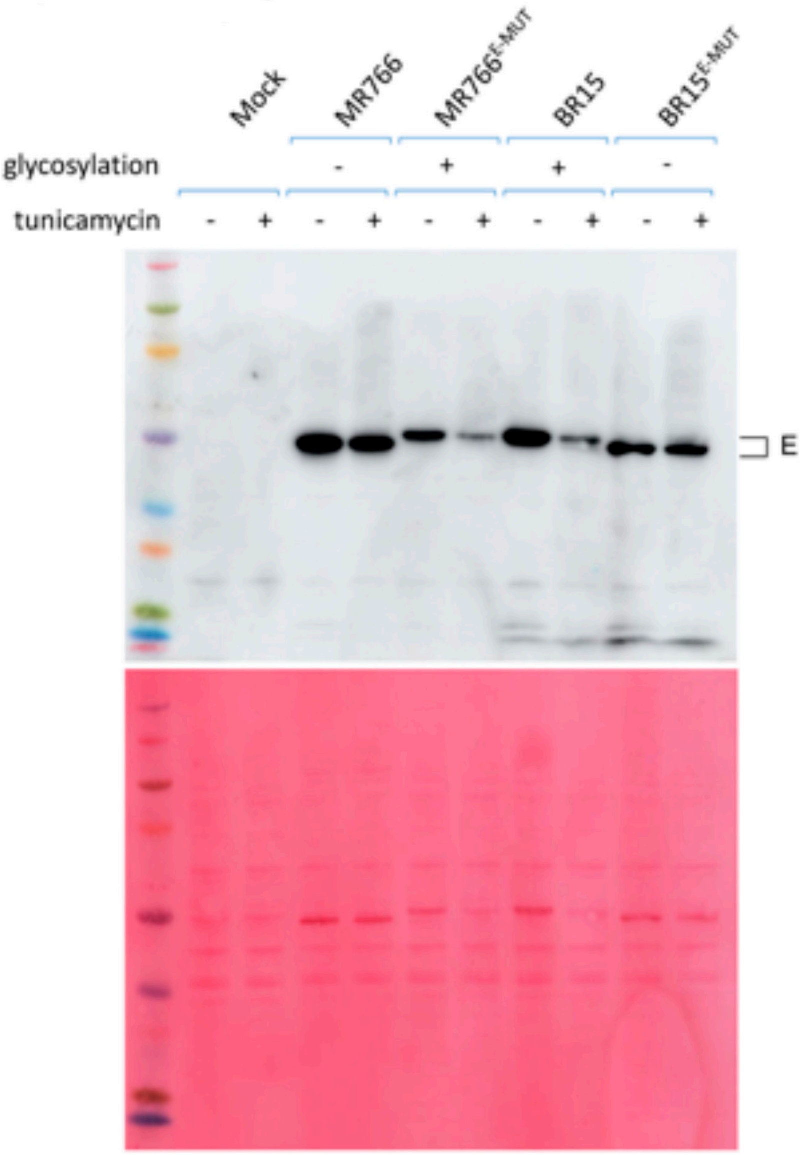

Figure S1: Tunicamycin treatment of Vero cells infected with ZIKV mutant molecular clones. Vero cells were infected with molecular clones at MOI of 1 and treated with tunicanycin, which is a well-known inhibitor of protein glycosylation. Cells were treated with 0.5 µg.mL^{-1} of tunicamycin 6 hours before lysis in RIPA buffer (24 hpi). Lysates were sonicated and quantified by BCA assay before been subjected to an immunoblot in non-reducing conditions. E protein was targeted with anti-EDIII rabbit polyclonal antibody. Upper panel represents anti-EDIII immunoblot and lower panel controls protein loadings (Ponceau staining). In these conditions, only glycosylated viruses are affected by tunicamycin treatment.

a.

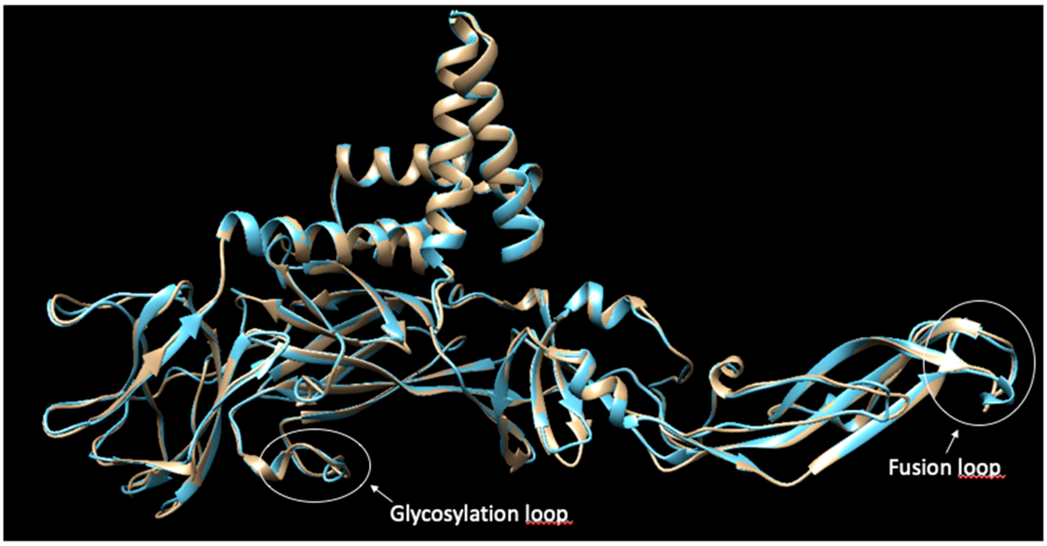

Blue = WT
Brown = MUT

b.

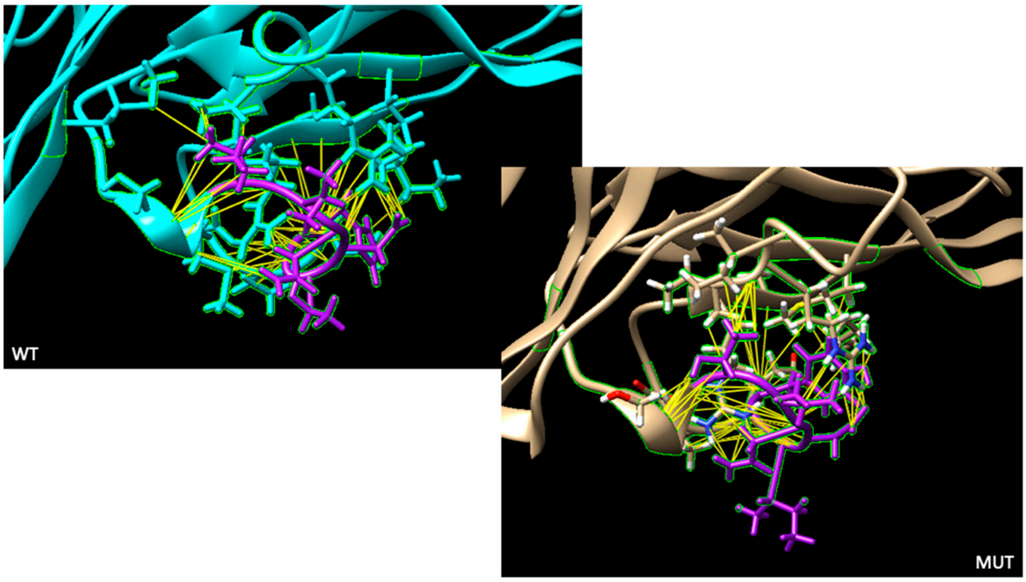

Figure S2: structures of ZIKV E wild-type and mutant proteins.
The sequences of BR15 and BR15E$^{\text{-MUT}}$ were submitted to I-TASSER (Iterative Threading ASSEmbly Refinement) Protein Structure and Functions Predictions server to generate PDB files. These files were used to compare WT and MUT structures with UCSF software Chimera. Chimera allowed a. to match structures and to identify regions of interest (glycosylation and fusion loop) and b. to highlight contacts with their surrounding regions.
